# Sampling the Mouse Hippocampal Dentate Gyrus

**DOI:** 10.3389/fnana.2017.00123

**Published:** 2017-12-12

**Authors:** Lisa Basler, Stephan Gerdes, David P. Wolfer, Lutz Slomianka

**Affiliations:** ^1^Division of Functional Neuroanatomy, Institute of Anatomy, University of Zürich, Zürich, Switzerland; ^2^Department of Pulmonology, University Hospital Zürich, Zürich, Switzerland; ^3^Neuroscience Center Zürich, University of Zürich, ETH Zürich, Zürich, Switzerland; ^4^Department of Health Sciences and Technology, ETH Zürich, Zürich, Switzerland

**Keywords:** dentate gyrus, stereology, volume, Cavalieri estimator, CE estimators, C57BL/6 mice

## Abstract

Sampling is a critical step in procedures that generate quantitative morphological data in the neurosciences. Samples need to be representative to allow statistical evaluations, and samples need to deliver a precision that makes statistical evaluations not only possible but also meaningful. Sampling generated variability should, e.g., not be able to hide significant group differences from statistical detection if they are present. Estimators of the coefficient of error (*CE*) have been developed to provide tentative answers to the question if sampling has been “good enough” to provide meaningful statistical outcomes. We tested the performance of the commonly used Gundersen-Jensen *CE* estimator, using the layers of the mouse hippocampal dentate gyrus as an example (molecular layer, granule cell layer and hilus). We found that this estimator provided useful estimates of the precision that can be expected from samples of different sizes. For all layers, we found that a smoothness factor (*m*) of 0 generally provided better estimates than an *m* of 1. Only for the combined layers, i.e., the entire dentate gyrus, better *CE* estimates could be obtained using an *m* of 1. The orientation of the sections impacted on *CE* sizes. Frontal (coronal) sections are typically most efficient by providing the smallest *CE*s for a given amount of work. Applying the estimator to 3D-reconstructed layers and using very intense sampling, we observed *CE* size plots with *m* = 0 to *m* = 1 transitions that should also be expected but are not often observed in real section series. The data we present also allows the reader to approximate the sampling intervals in frontal, horizontal or sagittal sections that provide *CE*s of specified sizes for the layers of the mouse dentate gyrus.

## Introduction

Design-based stereological methods provide easily interpretable and statistically valid estimates on the volumes, surfaces, lengths or numbers of regions or objects of interest in the brain. These methods have however been rather resilient to automation (e.g., Schmitz et al., 2014). They therefore often require more time to perform than methods that generate less reliable, more difficult to interpret, but, alas, just as publishable data. To minimize the time/workload one can adjust the precision of the estimates to the requirements of a study. It does not make sense to generate very precise estimates in the individual subjects that form, e.g., control and experimental groups, if the biological variability between subjects is high. Aside from the difference in the group means, group variances and the number of subjects determine the outcomes of statistical comparisons. If a high precision of estimates has little impact on the group variance, it may be less laborious to detect a group difference by relaxing precision but instead increasing the number of subjects in the groups (Gundersen and Østerby, 1981; West, 2012). To decide on the most efficient investment of work, one needs to be able to numerically assess the impact of estimate precision on group variance. While the group variance is part of the output of standard statistics, estimate precision is not. There are two ways to obtain it.

The estimation procedure can be replicated multiple times in a subject. The variance of the replicates, often expressed as the coefficient of variation (*CV*; standard deviation of the replicates divided by the mean of the replicates) would be an expression of precision of the estimates. While it may not be feasible to independently resample a structure, which would, e.g., require re-sectioning, an oversampling-subsampling approach can be used (Gundersen and Jensen, 1987). A very large or even exhaustive sample can be split into subsamples using multiple sampling intervals. This approach is quite labor intensive, and it has been used mostly to provide real life examples (Gundersen and Jensen, 1987; Roberts et al., 1993; McNulty et al., 2000; Slomianka and West, 2005) for the usefulness of the second way in which estimates of sampling induced variance can be obtained, i.e., mathematical estimators of precision.

Mathematical estimators of the coefficient of error (*CE*) of an estimate (e.g., Gundersen-Jensen estimator: Gundersen and Jensen, 1987; Gundersen et al., 1999; split-sample estimator: Cruz-Orive, 1990; Cruz-Orive and Geiser, 2004) can be based on the data from just one estimate, i.e., without the necessity of multiple replications of each estimate. The mean *CE* of the estimates should equal the *CV* of replicates. The facility of the *CE* calculation comes at a price. One needs to know or judge a variable critical to the calculation of the *CE*, the smoothness (*m*) of the dataset. Stereology software packages typically provide *CE* calculations for *m* values of 0 or 1. An *m* of 0 will provide a conservative estimate of precision, rarely exceeded in applications (Slomianka and West, 2005; Azim et al., 2012), but it may also be many-fold larger than the estimate provided by an *m* of 1. A second disadvantage is that precision can only be judged retrospectively, i.e., after datasets have been collected from the subjects.

While the labor associated with an oversampling-subsampling approach may not appear justified for one-off studies, our group has had a long-standing interest in the quantitative morphology of the hippocampus region and, in particular, the dentate gyrus (e.g., van Dijk et al., 2016). The work described here was performed to allow us to prospectively choose sampling intervals that provide *CE*s of a specified size. We here share the outcomes to allow readers to improve their qualified guesses at sampling schemes that provide the precision of estimates necessary in their studies and/or to select the *m* appropriate for the calculation of *CE* estimates.

## Materials and methods

### Tissue preparation

Two 17-weeks old female C57/Bl6 mice were used in this study. Animals were deeply anaesthetized with sodium pentobarbital (50 mg/kg) and perfused transcardially with 100 ml 4% paraformaldehyde in 0.13 M phosphate buffer (pH 7.4). All procedures were conducted in accordance with the Swiss animal welfare guidelines and approved by the cantonal veterinarian office of Zürich, Switzerland. The brains were dissected, split into left and right hemispheres using a razor blade, and post-fixed overnight.

Hemispheres were dehydrated in a graded series of alcohols and embedded in glycolmethacrylate (GMA; Technovit 7100, Heraeus Kulzer GMBH, Wehrheim, Germany) following the manufacturer's instructions, but using infiltration times of one day for each infiltration step. Sections were cut at a nominal thickness of 20 μm on a rotary microtome using steel knives. Prior to the cutting of each section, the block surface was wetted with water to soften the GMA. Care was taken to work as uniformly as possible and without interruption of the cutting of each hemisphere. One hemisphere from each brain was cut frontally; the remaining two hemispheres were cut either sagittally or horizontally. All sections were individually collected in well-plates, mounted on clean glass slides and oven-dried at 70°C for 1 h prior to staining.

Sections were Giemsa stained following the protocol of Iñiguez et al. (1985) by immersion in 25 ml stock solution (Merck, Darmstadt, Germany) diluted in 225 ml 67 mM KH_2_PO_4_ buffer for 40 min. After staining, sections were differentiated for 10 s in 1% acetic acid, dehydrated for 10 s in 96% ethanol followed by 10 min each in 99% and 100% ethanol. Sections were cleared in Histoclear and mounted with Histomount (Amresco, Solon, OH).

### Quantitative procedures on real sections

**Figure 1:**
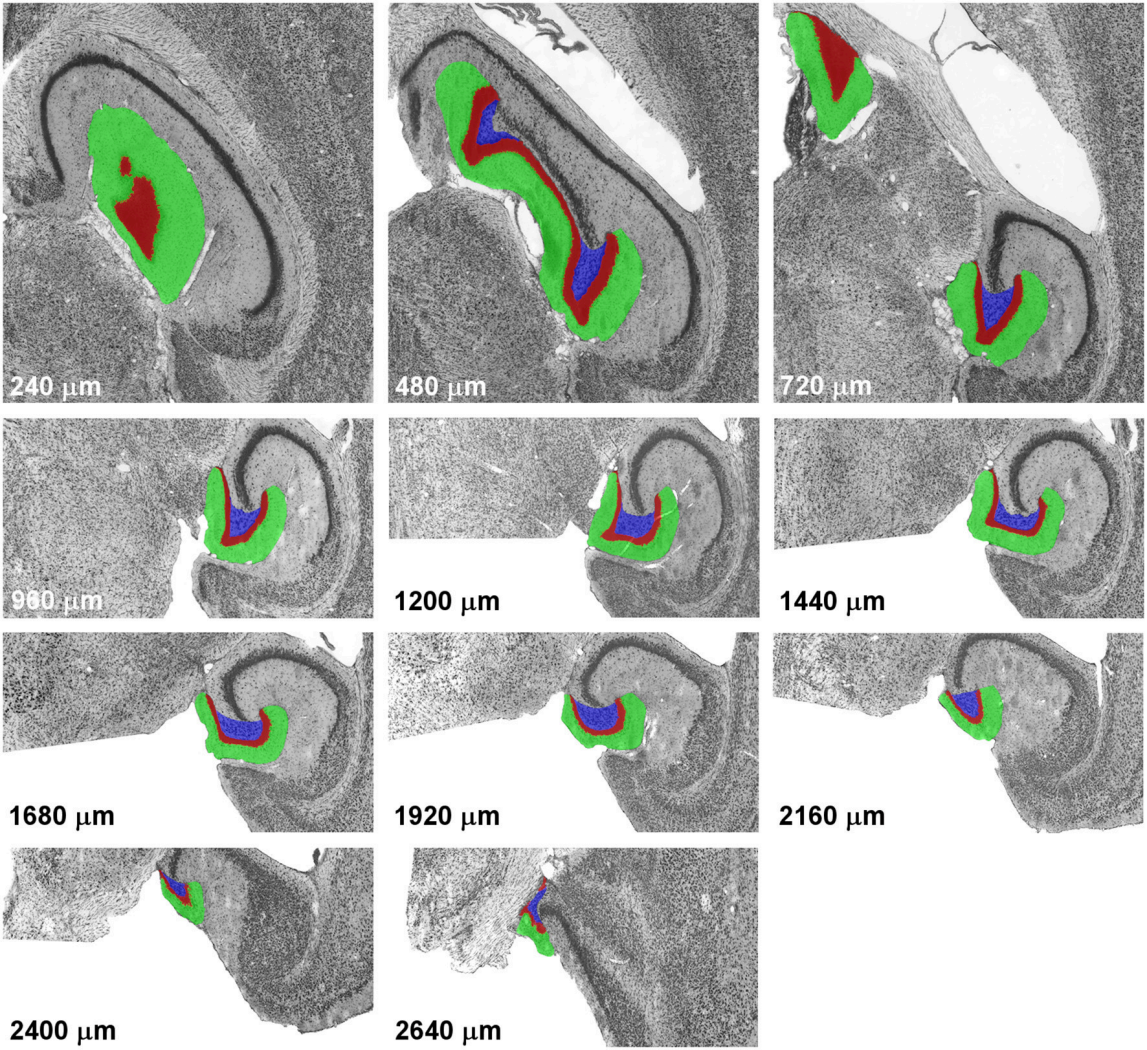
Definitions of the dentate gyrus layers. Illustrations represent a complete sample of every 12th section of a horizontal series passing through the mouse dentate gyrus from dorsal to ventral. With the exception of the last image, alignment is maintained, with the images of levels from 960 to 2,400 μm corresponding to the lower half of the images of more dorsal levels. The dentate molecular layer is highlighted in green, the granule cell layer in red and the hilus in blue.

The Cavalieri estimator (Gundersen, 1986; Gundersen et al., 1988) was used to generate the point counts in the datasets to be analyzed. Grids of points separated by 35 μm along the x- and y-axes were overlaid each section that contained the dentate gyrus using Stereoinvestigator software (MBF Bioscience, Williston. VT). Using this grid we obtained counts of around 10,000 points for the dentate granule cell layer, and the same grid was used for the hilus and molecular layer. The number of points in all three layers of the dentate gyrus is much higher than recommendations (up to a few hundred) but does not require much effort using modern stereological software. The high number of points in each section also minimizes the within-section variance, *S*^2^, that originates from slight variations in the placement of the grid and, therefore, slight differences in the counts that can be obtained in a section. The definitions of the dentate layers corresponded to those used by, e.g., (Haug, 1974), West et al. (1978) or Slomianka and Geneser (1993) and are illustrated along the septotemporal axis of the horizontal series in Figure 1.

### 3D modeling and analysis

An exhaustive series of horizontal sections of the entire hemisphere was digitized at a resolution of 3 μm/pixels. Images were aligned manually using gross features of the hemisphere and thereafter cropped to only include the hippocampal formation. The cropped images were manually fine-aligned using landmarks within the hippocampus at high magnification (Autoaligner 6.0.0, Bitplane, Schweiz). Thereafter, the layers of the dentate gyrus were highlighted in red, green or blue. Color channels were exported, and the dentate layers were reconstructed in 3D using Imaris 6.3.1 (Bitplane AG, Switzerland). A Gauss filter with a filter width of 20 μm was applied to the model to smoothen the edges representing individual section. While the very mild filtering did not remove all edges, wider filter settings did obliterate anatomical detail by, e.g., filling in part of the narrow space between the blades of the granule cell layer occupied by the hilus septally or by blunting the narrow extensions of the hilus beneath the ends of the blades of the granule cell layer. The surface of the model was calculated using a walking cubes algorithm and saved for further analysis.

Point counts were generated from the 3D models using the Imaris extensions and interface to Matlab. Models were sectioned frontally, horizontally and sagittally using sections of 1 μm thickness. Points were spaced at x- and y-distances that corresponded to the resolution of the original scans and/or the distance between the sections that were used to generate the models, i.e., 3 μm along the x- and y-axes when the models were sliced horizontally and 3 and 20 μm when the models were sliced frontally or sagittally.

### *CE* estimation

The Gundersen-Jensen *CE* estimator (Gundersen et al., 1999; see formula below) was used to estimate the *CE*s for all possible samples up to sampling intervals of 20 for the real sections and up to 400 for the virtual sections of the 3D models. The maximum spacing between samples was therefore 400 μm for both real and virtual sections. *CE*s were calculated for an *m* of 0 and 1.

$$\begin{array}{l} CE\;:=\frac{\sqrt{\left(3\left(A-S^{2}\right)+C-4B\right)\times \alpha +S^{2}}}{\sum P} \end{array}$$

in which

$A=\sum_{i=0}^{n}\left({P_{i}}^{2}\right)$, i.e., the sum, across all sections of the sample, of the counts in each individual section (*P*_*i*_) squared

$B=\sum_{i=0}^{n}\left(P_{i}\times P_{i+1}\right)$ i.e., the sum, across all sections of the sample, of *P*_*i*_, multiplied by the counts in the following section of the sample, i.e., *P*_*i*+1_, and

$C=\sum_{i=0}^{n}\left(P_{i}\times P_{i+2}\right)$ i.e., the sum, across all sections of the sample, of *P*_*i*_ multiplied by the counts obtained in the next to the following section, i.e., *P*_*i*+2_

α is 1/12 for an *m* = 0 and 1/240 for *m* = 1

Σ*P* is the sum of the points counted in all section.

$$\begin{array}{l} S^{2}\;:=0.0724\times \frac{\bar{b}}{\sqrt{ā}}\times \sqrt{n\times \sum P} \end{array}$$

in which

$\frac{\bar{b}}{\sqrt{ā}}$ is a shape factor that can be calculated from the boundary length, *b*, and area, *a*, of the dentate layers. In that the contribution of *S*^2^ (also referred to as noise, local error or nugget variance) to the *CE* is minimal using the point counts obtained in this study, we did not estimate the shape factor, but used the nomogram in Gundersen and Jensen (1987) to select 10 as a conservative estimate of $\frac{\bar{b}}{\sqrt{ā}}$,

*n* is the number of sections contained in the sample, and

Σ*P* is the sum of the points counted in all sections.

The *CE*s were estimated empirically by calculating, for each sampling interval from 2 to 20, the coefficient of variation of all samples belonging to a sampling interval. E.g., for the sampling interval 17, 17 samples are generated. Sample 1 contains sections 1, 18, 35, 52 … etc., sample 2 containing sections 2, 19, 36, 53 … etc., continuing up to sample 17 which contains section 17, 34, 51, 68 etc. A volume estimate is calculated for each of the 17 samples. The coefficient of variation for the sampling interval 17 is finally calculated by dividing the standard deviation of the 17 possible estimates by the mean of the 17 estimates.

Estimates of the *CE* for both *m* values and empirical estimates were plotted against sampling intervals.

## Results

### Empirical *CE*s and estimator *CE*s obtained from the sectioned dentate gyrus

113 (frontal series), 130 (horizontal series), or 107 (sagittal series) sections containing the dentate gyrus were obtained for analysis. No sections were missing. In these series, we obtained point counts of 5,284 to 5,589 for the hilus, 9,956 to 11,927 for the granule cell layer, and 28,854 to 30,674 for the molecular layer.

**Figure 2:**
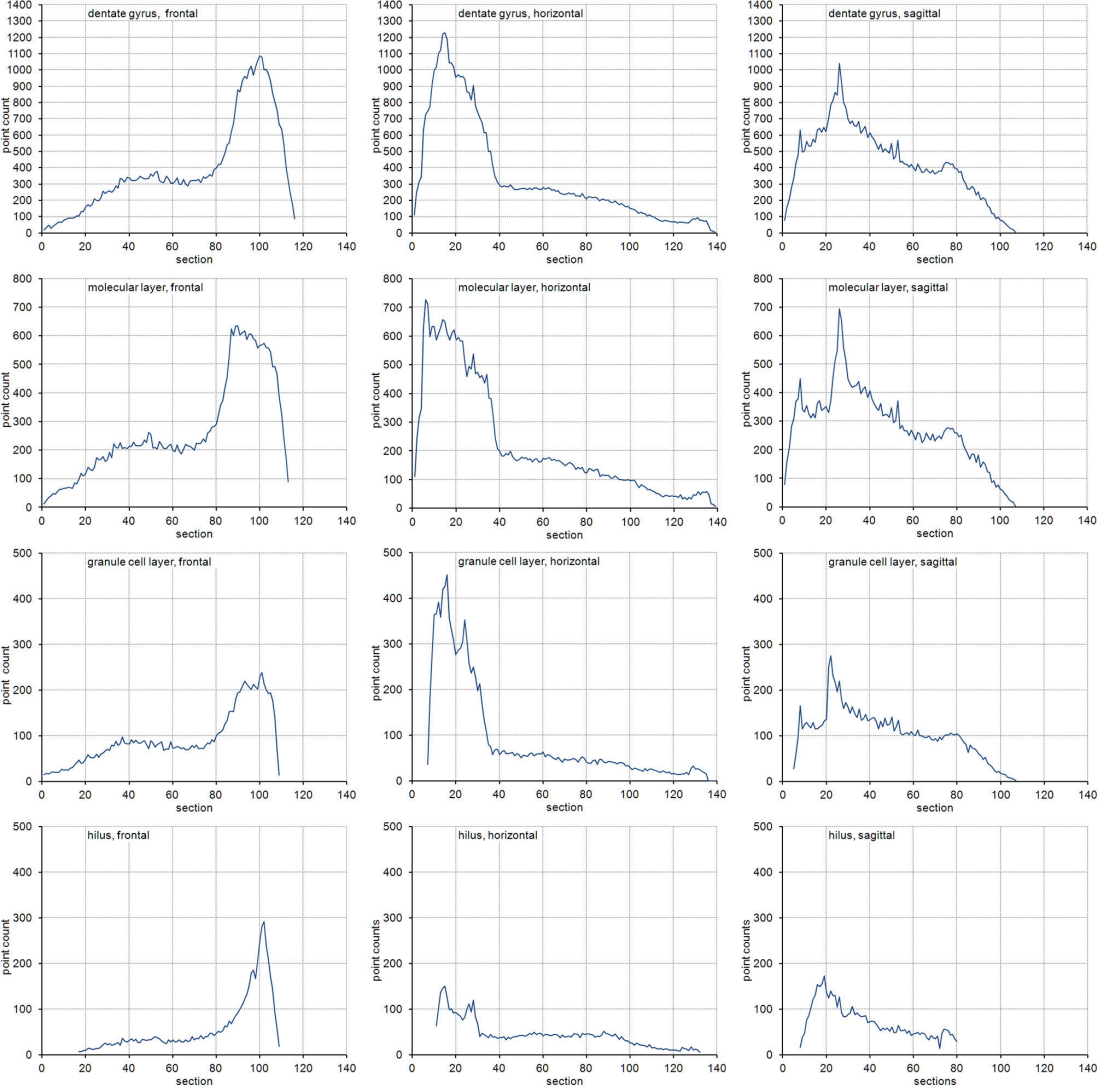
Volume distributions of the dentate gyrus and its layers. Volume distributions are illustrated by the point count obtained in each section. Cutting directions are anterior to posterior for frontal sections, dorsal to ventral for horizontal sections and medial to lateral for sagittal sections.

**Figure 3:**
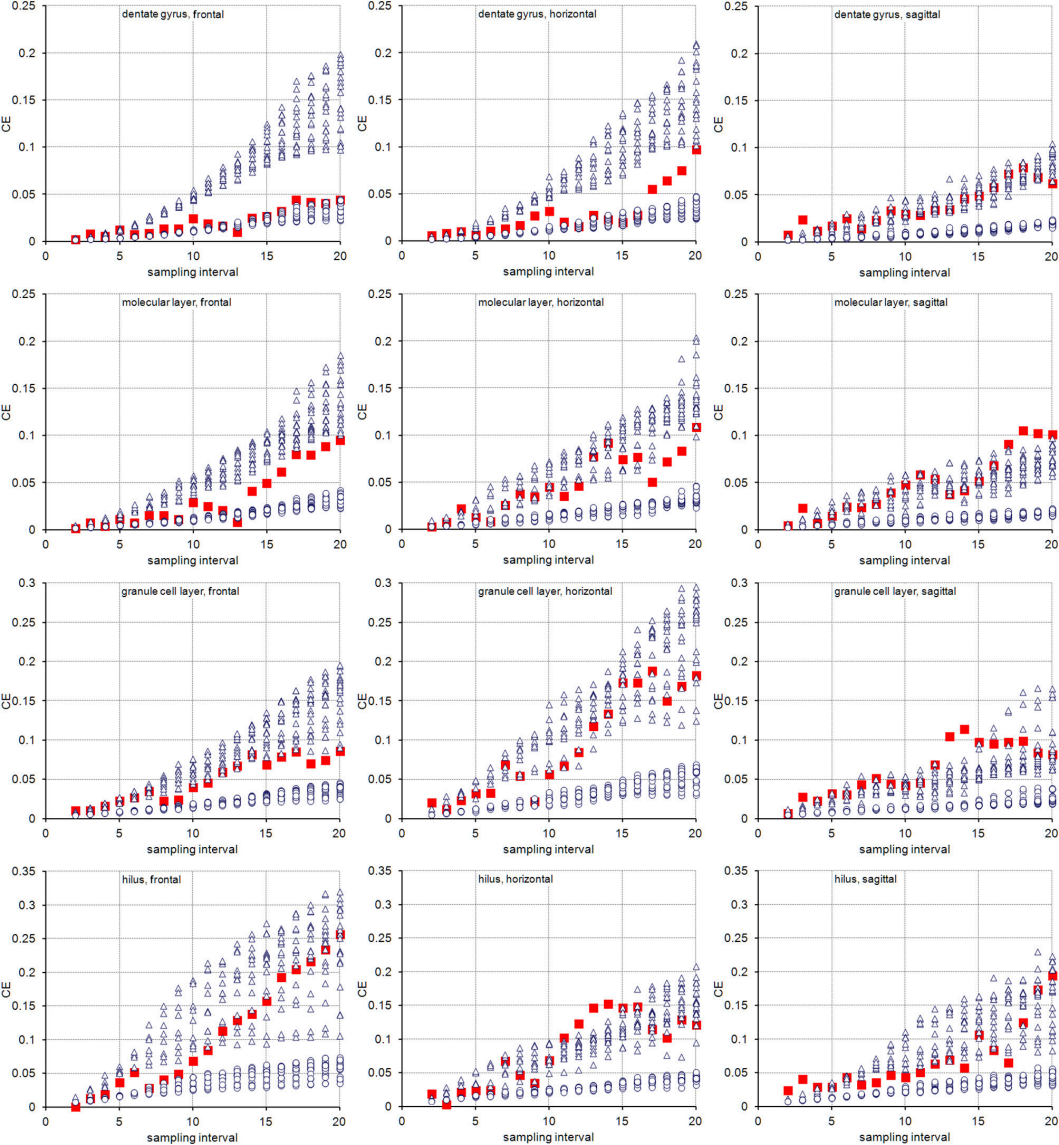
Measurement precision vs. sampling frequency. *CE*s are plotted for increasing intervals between the sampled sections. Empirical estimates of the *CE*s (filled squares) are typically bounded by Gundersen-Jensen *CE* estimates for *m* = 0 (open triangles) and *m* = 1 (open circles). Sampling intervals at which the empirical *CE* exceeds the range of estimated *CE*s correspond to intervals between peaks in the volume distributions of the layers (Figure 2). How many sections will be analyzed depends on the size of the structure of interest along the direction of the cutting. E.g., about 110 frontal 20 μm thick sections would contain the granule cell layer (see Figure 2). Series sampled using an interval of 10, i.e., every 10th section is collected (200 μm between sections), would contain 11 sections to be analyzed. If sections are cut, e.g., 40 μm thick, a series of every 5th section (200 μm between sections) would generate a similar number of sections.

The volume distributions of three layers of the dentate gyrus along the direction of cutting are illustrated in Figure 2. The empirical *CE*s (*CV*s of replicates), belonging to subsamples up to a sampling interval of 20 (i.e., using every 20th section), are illustrated in Figure 3 together with the *CE* estimates of each subsample of each sampling interval using an *m* = 0 or an *m* = 1. In addition, Figure 3 provides these data for the entire dentate gyrus, i.e., the collated data of the hilus, granule cell layer and molecular layer.

For the layers of the dentate gyrus, *CE* estimates using both *m* values typically provide upper and lower bounds for the empirical *CE*s obtained. Exceptions were found for sampling intervals around 14 for the granule cell layer and around 18 for the molecular layer in the sagittal series and sampling intervals around 14 for the hilus in the horizontal series. Around these intervals, the empirical *CE*s exceeded the estimates. At sampling intervals larger than 10, the empirical *CE*s of the hilus and granule cell layer were usually found within the scatter of *CE* estimates that used an *m* = 0, i.e., *CE* estimates using an *m* = 0 are better predictors of the empirical *CE*s. For lower sampling intervals, the small differences between the *CE* estimates resulting from different *m* values and the limited number of subsamples available to calculate empirical *CE*s make it difficult to evaluate which *m* would result in a better *CE* estimation.

The entire dentate gyrus behaved somewhat differently from its layers. For all sampling intervals used in frontal sections and most sampling intervals smaller than 15 in horizontal sections, *CE*s that were estimated using an *m* = 1 were better predictors of the empirical CEs. In sagittal series, an *m* = 0 again provided better predictions.

We did not estimate the shape factors that are needed to estimate the contribution within-section variance, *S*^2^, to the *CE* estimates, but instead used a conservative shape factor of 10. The high number of points counted in each section should result in a minimal contribution of *S*^2^ to the *CE*s. We also calculated *CE*s using an unrealistically high shape factor of 50, which barely generated perceptible changes in the graphs (data not shown).

### Empirical *CE*s and estimator *CE*s obtained from reconstructions

**Figure 4:**
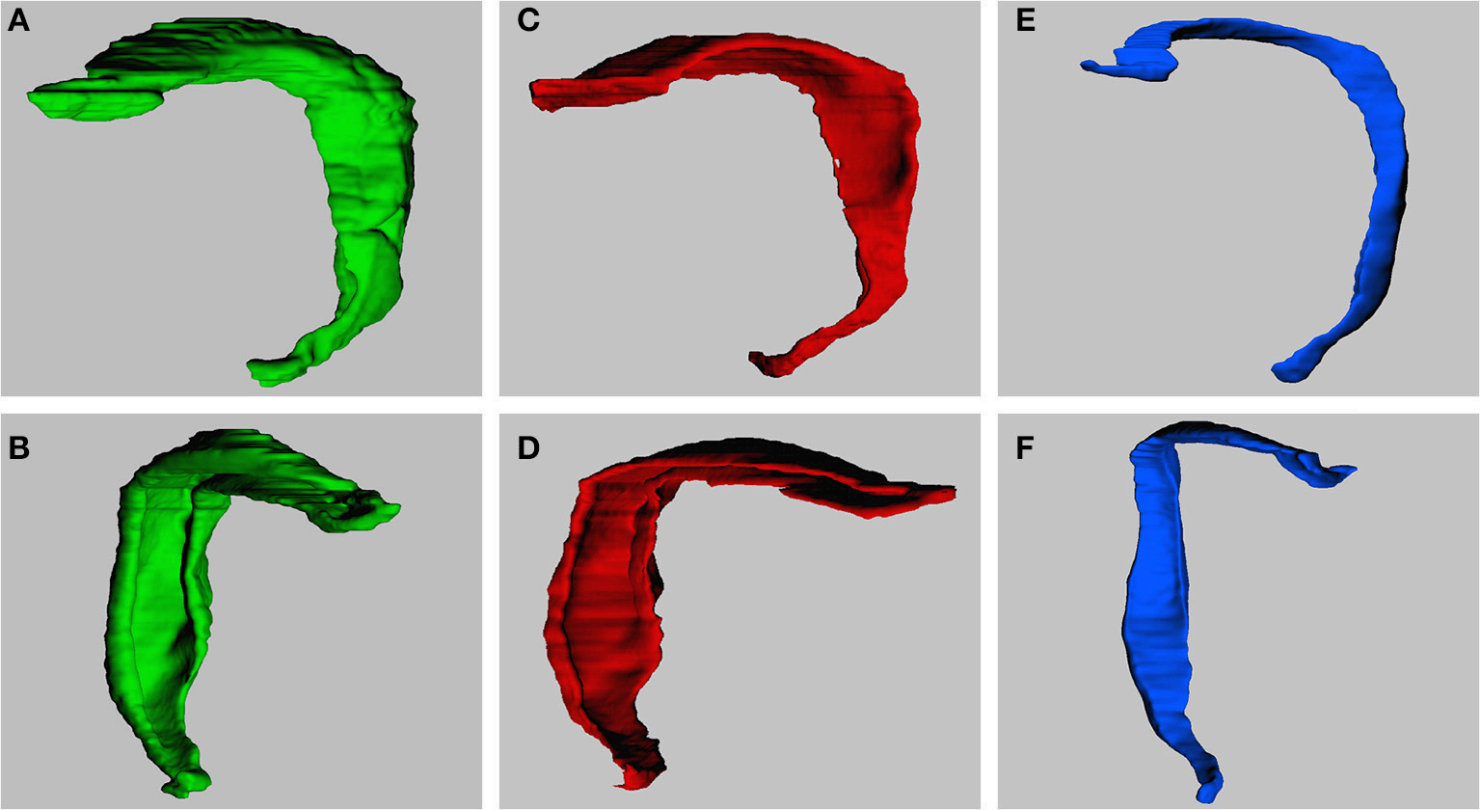
3-Dimensional models of dentate gyrus layers. Models are constructed based on the horizontal series of real sections. Models represent the dentate molecular layer **(A,B)**, the granule cell layer **(C,D)** and hilus **(E,F)**. Views are from medial to lateral **(A,C,E)** or into the concavity of the dentate layers from anterior to posterior (**B,D,F**; slight variations between exact angles).

Figure 4 illustrates the 3D models that were obtained after reconstructing the dentate layers from a horizontal series of sections. The volume distributions obtained from virtually sectioning the models frontally and sagittally largely corresponded to the volume distributions seen in the real sections.

The comparison of empirical and estimated CEs was performed analogous to the comparison in real sections, but using much thinner, 1 μm thick virtual sections. Sampling intervals up to 400 were assessed, at which interval the distance between the virtual sections corresponded to the distance between the 20 μm thick real section and a sampling interval of 20 (20 × 20 μm = 400 μm)—the maximum used to assess the real sections. The decrease in section thickness provided larger numbers of subsamples for sampling intervals with low section to section distances.

**Figure 5:**
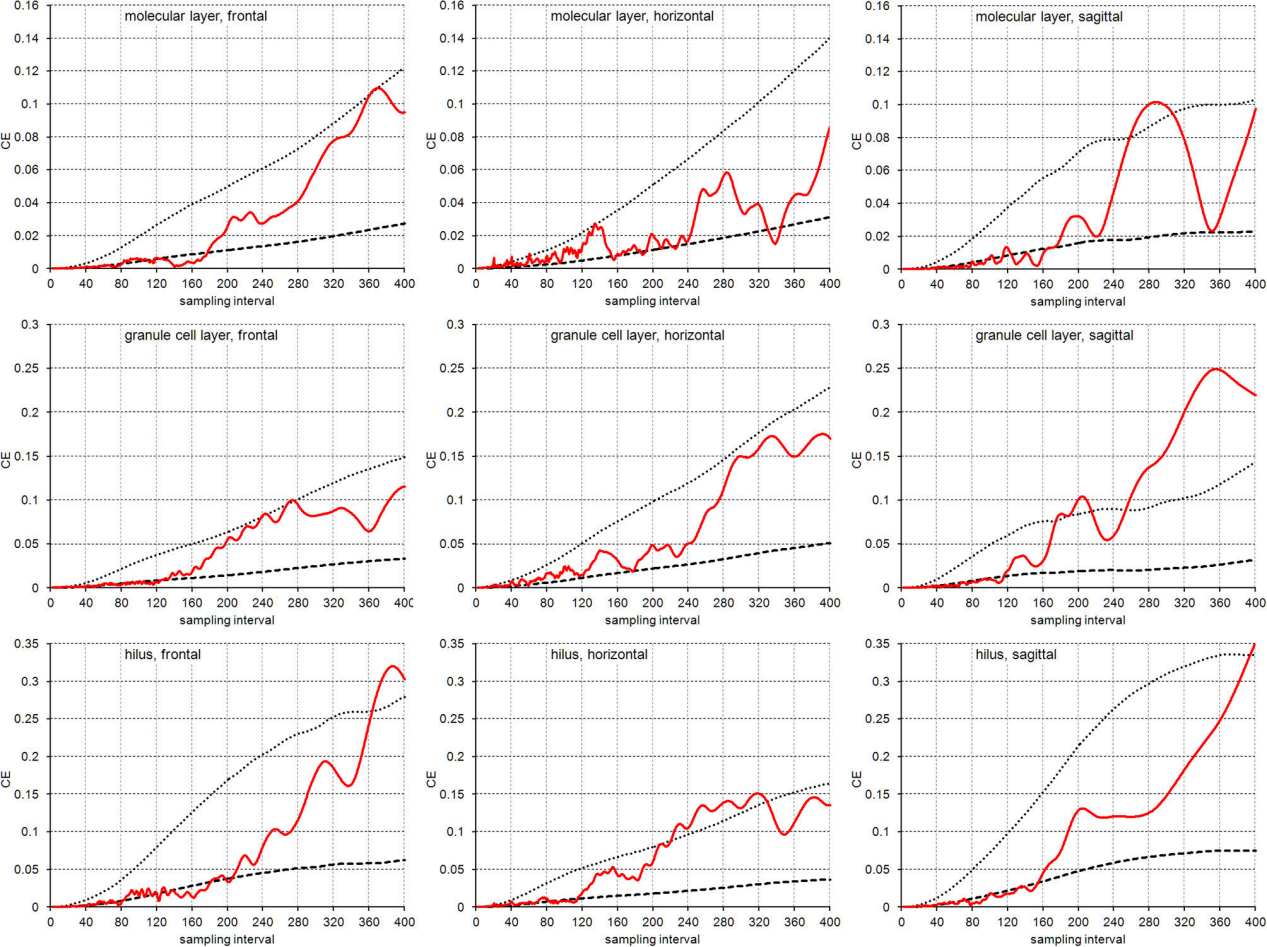
CE estimates based on virtual sectioning of 3D models. Empirical *CE* estimates (solid lines) are typically bounded by the means of the Gundersen-Jensen *CE* estimates for *m* = 0 (dotted lines) and *m* = 1 (broken lines) and exceed *CE* estimates for sampling intervals that correspond to the distance between peaks in the volume distributions (Figure 2). The very intense sampling (1 μm sections) and higher subsample numbers for low sampling intervals show transitions in the behavior of the empirical *CE* from following an *m* = 1 at low sampling intervals to an *m* = 0 at high sampling intervals. In addition, empirical *CE*s now oscillate with increasing amplitude and period for increasing sampling intervals (Zitterbewegung; best seen in the frontally sectioned hilus).

Figure 5 illustrates the empirical *CE*s obtained from the virtual sections together with the means of the *CE* estimates obtained from the subsamples of each sampling interval using an *m* = 0 or an *m* = 1. Again, the empirical *CE*s are typically bounded by the estimated *CE*s that were calculated for the two *m* values. For sampling intervals above 200, corresponding to sampling every 10th real section, the outcomes of sampling virtual sections largely correspond to the sampling of real sections. Instead, for sampling intervals lower than 120, corresponding to the sampling of every 6th section, *CE*s are usually better predicted by an *m* = 1. For the intervening intervals, the quality of the prediction of different *m* values depends on the layer of the dentate gyrus and the orientation of the sections.

In addition, we observed the Zitterbewegung of the empirical *CE* estimates, i.e., oscillating changes in the size of the *CE* estimate that increase in amplitude and period with increasing sampling intervals (best seen in the frontally sectioned hilus in Figure 5).

## Discussion

In summary, frontal sections of the dentate gyrus are the most efficient way to generate quantitative estimates of the volumes of its layer. For a given sampling interval, *CE* values are generally lower for the granule cell layer and molecular layer in frontal sections than those obtained from sagittal or horizontal sections. Also, the number of sections to be cut frontally is lower than those that need to be cut horizontally. Finally, the layers are quantitatively “better behaved” in frontal than in sagittal sections, in which the *CE* for some sampling intervals may be difficult to predict using the Gundersen-Jensen estimator (see also below). An *m* of 0 would be the appropriate choice for *CE* estimations. If the entire dentate gyrus is the region of interest, frontal sampling is exceedingly efficient and provides volume estimates with *CE*s of less than 5% assessing as few as 5 to 6 sections. Efficiency is, of course, not the only factor to be considered when a direction to section the material is chosen. Another factor would be the ability to define interregional and interlaminar boundaries (Slomianka and West, 2005).

### Extrapolation to other estimators

With some caution, it should be possible to extrapolate the efficiencies seen for volume estimates to estimators of number, length and surface. Although differences in the densities and morphologies of cells are present in the dentate gyrus, changes along the hippocampal axes are generally modest and gradual (Gaarskjaer, 1978; Jinno et al., 1998; Uchida et al., 2005; Jinno and Kosaka, 2010; Jinno, 2011; Amrein et al., 2015; Buckmaster et al., 2017). Estimators of parameters other than volume are therefore likely to generate distributions along the direction of cutting that resemble the volume distributions shown here. Note that the contribution of within-section variance, *S*^2^, to the *CE* is calculated very differently from that of point counts used in volume estimations. It equals the sum of interactions between stereological probe and the parameter of interest (Gundersen et al., 1999), e.g., the number of intersections between a test area and capillaries or cell processes in estimations of their lengths (Løkkegaard et al., 2001; Nykjær Nikolajsen et al., 2011; Gondré-Lewis et al., 2016) or the number of intersections between test lines and cortical or neuronal surface (Acer et al., 2010; Loesch et al., 2010). The contribution of *S*^2^ to the *CE* ($CE_{S^{2}}$) would be $\sqrt{sum\;of\;interactions}/sum\;of\;the\;interactions$, i.e., 0.1 (or 10%) for a count of 100. While point counts for volume estimates can be easily increased to make *S*^2^ negligible, this may not be the case for other types of probe-feature interactions. Using our data on volume estimate precision, a rough guesstimate of the *CE*s to be expected from estimators of number, length or surface, could be $\sqrt{C{E_{vol}}^{2}+C{E_{S^{2}}}^{2}}$. If, e.g., the granule cell layer is sampled in frontal sections spaced at 200 μm intervals (sampling interval 10, *CE*_*vol*_ ~ 0.04) and if, e.g., a total of 200 cells is counted in this sample of sections, one may expect a *CE* of ~ $\sqrt{0.04^{2}+0.07^{2}}$ or 0.08. The small increase of from 0.07 to 0.08 may suggest that the selection of sections does not have a major impact on the final *CE*, but it actually contributes one quarter to the variance generated by the sampling (0.04^2^ / 0.08^2^ = 0.25). Also note that counting very many cells does not guarantee a small *CE*. One may decide to count 10,000 granule cells in a sample of every 20th section. Even though the $CE_{S^{2}}$ only amounts to 0.01, one would still have to expect a final *CE* ranging from ~0.08 (sagittal or frontal sections) to 0.18 (horizontal sections) because of the selection of only every 20th section. Clearly, counting 200 cells in samples from every 10th section is a massively more efficient way to obtain a *CE* of 0.08.

A rough guesstimate of the *CE* will provide a starting point for a study. Once a data set is available, *CE*s should, of course, be estimated using the approaches appropriate for the method that has been selected. Such estimates are typically provided by stereological software packages. Calculated examples of *CE* estimations can be found in, e.g., West et al. (1991) and (West, 2012) for estimates based on fractionator sampling or in West and Gundersen (1990) if estimates are based on separate density and volume estimates.

### Which smoothness factor to choose?

While an estimate of the smoothness factor, *m*, also can be calculated (Kiêu et al., 1999), datasets available from typical applications are too small to provide robust estimates (Cruz-Orive, 1999; Gundersen et al., 1999; García-Fiñana and Cruz-Orive, 2004). The selection of a smoothness factor *m* is therefore often a matter of investigator judgment. The original form of the Gundersen-Jensen *CE* estimator used an *m* of 0 (Gundersen and Jensen, 1987), while the later revision made an argument for the use of an *m* of 1 (Gundersen et al., 1999). We previously found that an *m* of 0 provided better estimates for the hippocampal CA1 pyramidal cell layer (Slomianka and West, 2005) and here confirm this observation for the layers of the dentate gyrus. The smoothing of the 3D models in excess of 20 μm filter width did hide anatomical detail. Even exhaustive series of 20 μm thick sections do not appear to provide sufficient resolution to predict all anatomical features from section to section, which would be a justification for the choice of an *m* of 0. Another possible explanation for larger than expected *CE*s at low sampling intervals are sources of variance not accounted for by the Gundersen-Jensen *CE* estimator. One source may be observer error, i.e., variations in counts that result from observer uncertainty about the location of the boundary of the structure that is being assessed. Another source may be variance of the exact distances between the sections and their thicknesses (Baddeley et al., 2006; Ziegel et al., 2010). Both types of error have the potential to significantly increase *CE*s, in particular when sampling intervals are small. In contrast, one μm thick virtual sections of 3D models, which retained the anatomical detail, do provide the necessary resolution and provide some sampling intervals that allow the use of an *m* of 1 also for the dentate layers. Such sampling intervals would, however, be prohibitive in terms of the workload required in the real world. Also, *CE*s that are generated by such intervals are so low (usually 0.02 or less) that the workload is unlikely to be justified considering that animal to animal variation is usually much higher. In statistical comparisons, the chances to observe group differences would be more efficiently increased by increasing the number of subjects (Gundersen and Østerby, 1981; West, 2012).

### Cases of poor *CE* estimates

Most stereological protocols encompass the selection of sampling sites at regular intervals along the x- and y-axes of the section. *CE* estimators may perform poorly if there is a match of sampling intervals with periodic changes in anatomy. Such changes may relate to repeated units in the organization of the brain, e.g., cortical barrels or columns. Even though a perfect match is unlikely to occur, it is relatively easy to avoid in the plane of the section by the random application (including rotation) of the grid of sampling sites. There is no similar way to alter sampling positions along the z-axis, and matches between sampling interval and periodic changes in morphology will result in poor *CE* estimates. When empirical *CE*s exceeded *CE* predictions, the associated section sampling intervals were close matches to the distance between two prominent peaks in the volume distribution the dentate layers. The peaks in turn reflect sagittal or horizontal sections that pass through large parts of the suprapyramidal and, once again, the infrapyramidal blades of the dentate gyrus layers. “Crest-on” frontal sections avoid this from happening. If horizontal or sagittal sections are preferred for reasons unrelated to estimate precision, the respective intervals should be avoided.

With these few and avoidable exceptions, the Gundersen-Jensen *CE* estimator provides useful bounds for the precision to be expected from sampling schemes of the mouse dentate gyrus.

## Author contributions

LB and SG performed the practical work under the supervision of DW and LS. LS wrote the first draft of the manuscript, which was revised in collaboration with all co-authors.

## Disclosure

The data presented in this manuscript were part of theses written by LB and SG in fulfillment of the requirements to obtain the degree Dr. med. at the University of Zürich.

### Conflict of interest statement

The authors declare that the research was conducted in the absence of any commercial or financial relationships that could be construed as a potential conflict of interest.
